# Alternating Current Stimulation Promotes Healing of Bone Fractures in Rabbits

**DOI:** 10.3390/bioengineering12121356

**Published:** 2025-12-12

**Authors:** Shaohui Geng, Hesong Wang, Guiyang Huo, Li Wang, Haixu Jiang, Heng Xu, Enfan Xiao, Li Liu, Xingjian Wang, Xia Li, Guangrui Huang, Xiaohong Mu, Anlong Xu

**Affiliations:** 1 School of Life Sciences, Beijing University of Chinese Medicine, 11 Beisanhuandong Road, Chaoyang District, Beijing 100029, China; 2 Department of Rehabilitation, Jiangsu Province People’s Hospital and Nanjing Medical University First Affiliated Hospital, Nanjing 210036, China; 3 School of Rehabilitation Medicine, Nanjing Medical University, Nanjing 210029, China; 4 Wudang Medical Research Institute, Beijing University of Chinese Medicine, Beijing 100029, China; 5 Department of Orthopedic, Dongzhimen Hospital, Beijing University of Chinese Medicine, Haiyuncanghutong Road, Dongcheng District, Beijing 100700, China; 6 Hong Kong Institute of Advanced Studies, Sun Yat-Sen University, Hong Kong, China; 7 School of Life Sciences, Sun Yat-Sen University, Guangzhou 510275, China

**Keywords:** alternating current, fracture, bone defect, mechanism

## Abstract

Background: Bone fracture is a partial or complete break in the continuity of a bone, which poses a significant healthcare burden. It is important to discover a novel method to stimulate and speed-up the healing of bone fractures. Aim: This study aimed to investigate the effects and mechanisms of alternating current (AC) in promoting bone fracture healing. Methods: A rabbit bone fracture model was used. X-ray and Micro-CT evaluated fracture healing, while HE staining and immunohistochemistry assessed morphological changes. In vitro, pre-osteoblastic cells were tested with alizarin red S staining and alkaline phosphatase (ALP) activity. RNA-seq analysis explored potential mechanisms. Results: X-ray evaluation showed that alternating current stimulation (ACS) promoted bone formation and shaping by day 14 post-treatment. Micro-CT results revealed significant new bone formation as early as day 3 and day 7 (*p* < 0.05). HE staining indicated more trabecular bone formation in the ACS group compared to the model group at days 7 and 14. Immunohistochemistry showed higher expression of BMP-2 and VEGF in the ACS group by day 7. In vitro, ACS enhanced osteogenic differentiation, increasing calcified nodule formation and ALP activity. Gene expression analysis demonstrated significant changes in key osteogenic genes, confirmed by multiple immunohistochemical staining. Conclusions: ACS may be a novel method for treating bone fractures more rapidly, significantly relieving the patient’s burden, particularly in the early stages of bone healing.

## 1. Introduction

Bone fracture, which is a partial or complete break in the continuity of a bone, is a common medical problem with nearly 180 million new fractures every year [1]. Traumatic incidents and certain medical conditions, such as osteoporosis and cancer, can be causes of bone fractures [2]. Traumatic fractures lead to a substantial burden on healthcare systems worldwide. Fractures might cause health loss, decreased productivity, and work absence, leading to a serious economic burden [3]. Treatments of bone fractures include surgery and conservative therapy based on natural healing. Nowadays, surgery is the mainstream clinical treatment in dealing with bone fractures, but surgical methods have their own risks such as infection, deep vein thrombosis, and pulmonary embolism [4]. Thus, to speed up the healing of fractured bones and reduce productivity loss, it is of particular importance to discover a novel method to speed up the healing of bone fractures.

Electrical stimulation has been demonstrated to promote bone healing in previous studies [5,6]. Previous studies have primarily focused on modalities such as direct current (DC), capacitive coupling (CC), and pulsed electromagnetic field (PEMF). Besides electrical stimulation modalities, extracorporeal shockwave therapy (ESWT) has emerged as another prominent non-invasive physical therapy for promoting bone healing. For instance, Paterson et al. found that DC could significantly accelerate bone healing at four weeks in the experimental model, with a constant sum current of 20 μA for clinical union in dogs with fractures [7]. Similarly, Buch et al. demonstrated that 5 or 20 μA DC stimulation significantly enhances osteogenesis in a rabbit model of fractured bone, in which more bone mineral content was found after treatment [8]. Rossini et al. investigated if a CC electric field relieved pain in vertebral fracture patients [9], while Griffin et al. proved that electromagnetic field stimulation could benefit bone healing in treating the delayed union and non-union of long bone fractures after analyzing four randomized controlled trial studies involving 125 participants [10].

Despite these advances, existing electrical stimulation and ESWT technologies still have some limitations. DC stimulation often leads to charge accumulation and electrode degradation [11], which may cause tissue damage and limit long-term applicability. CC and PEMF, although non-invasive, suffer from inconsistent field distribution and limited penetration into bone tissue, reducing their efficacy in complex fracture geometries [9,12]. In contrast, alternating current (AC) stimulation offers several distinct advantages that address these shortcomings. Unlike DC, AC prevents net charge accumulation through periodic current reversal, minimizing the risk of tissue damage and electrode corrosion [13]. This feature allows for safer and more sustained application, particularly in sensitive or deep-seated fracture sites. Compared to CC and PEMF, AC enables more uniform and controllable electric field distribution within bone tissue, enhancing its ability to promote cellular activities such as osteoblast proliferation and differentiation [14]. Furthermore, compared to ESWT [15], AC provides continuous and adjustable stimulation without requiring specialized acoustic wave equipment. Moreover, AC stimulation can be finely tuned in terms of frequency and amplitude to selectively target cellular and molecular pathways involved in bone regeneration, providing a level of mechanistic specificity not achievable with conventional PEMF or CC. No mechanistic studies have tested the use of alternating current (AC) on bone fractures, although physicians in China have demonstrated the safety and efficiency of alternating current on bone fractures [16,17,18] in clinical practice.

In this study, we conducted a systematic investigation into the mechanism of AC stimulation in bone fracture repair using a rabbit model. While prior reports have indicated the therapeutic potential of AC, a comprehensive mechanistic understanding bridging physiological, cellular, and molecular levels remains to be fully established. Our work addresses this gap by providing multi-level evidence on how AC stimulation promotes osteogenesis. Fractures were induced using bone biting forceps, and AC was applied as the primary intervention. Healing progression was evaluated via X-ray and micro-CT imaging, while histological and immunohistochemical analyses were employed to assess morphological and molecular changes. Complementary in vitro experiments were performed to elucidate the underlying cellular and molecular mechanisms. The novelty of this study lies in its integrated approach, which systematically correlates AC stimulation with accelerated early-stage bone regeneration and delineates the associated dynamic changes in gene expression and key osteogenic factors, thereby establishing a mechanistic framework for its therapeutic application. Our work provides important mechanistic evidence supporting the use of AC stimulation for bone fracture healing, paving the way for its future clinical translation.

## 2. Methods

### 2.1. Animals

**Figure 1:**
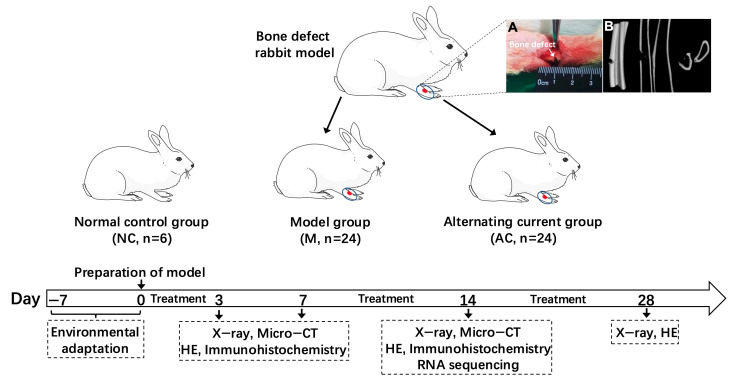
Flow chart of this study. (**A**) Surgical process photos of rabbit bone defect model; (**B**) Micro–CT photos of rabbit bone defect model.

A total of 54 New Zealand rabbits (male, clean grade, weighing 2.5–3 kg) were purchased from Beijing Long’an experimental animal breeding center. After acclimatization for 7 days, all animals were randomly divided into three groups: the normal control (NC) group (n = 6), bone-fractured model (M) group (n = 24), and alternating current (AC) treated group with fractured bones (n = 24). The animals were housed in a stable environment at a temperature of 20~25 °C and relative humidity of 40~50%, with a 12 h light/dark cycle. The experiments in this study were approved by the Ethic Committee of Animal Experimentation for Beijing University of Traditional Chinese Medicine. A flow chart of this study is shown in Figure 1.

### 2.2. Preparation of Bone-Fractured Model of Rabbit

The rabbits were injected intravenously into the ear margin with 3% sodium pentobarbital solution (30 mg/kg). A 2 cm longitudinal incision was made in the middle of the radius after skin preparation. A bone fracture was made by 3 mm-width bone biting forceps into the medullary cavity, avoiding important blood vessels and nerves. Antibiotics were dispersed at the fracture site after the operation.

### 2.3. Percutaneous AC Stimulation

**Figure 2:**
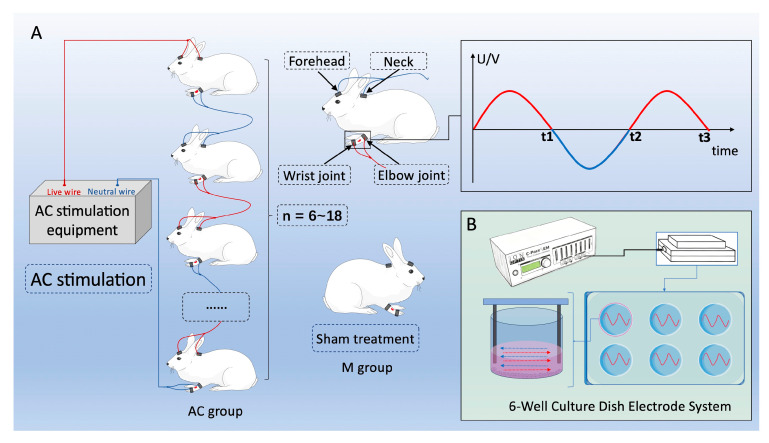
Schematic diagram of alternating current (AC) stimulation and sham treatment, illustrating the electric field distribution and current paths. (**A**) Schematic diagram of grouping protocol and waveform of AC treatment of fractured rabbits; (**B**) Schematic diagram of AC stimulation of MC3T3-E1 cells.

In the treatment group, the rabbits were fixed, and their forelimbs were exposed. The circuitry connection consisted of 2 electrode wires (4 electrode patches) per rabbit. Two electrode patches were connected around the skin of the wrist joint and the elbow joint. Another two patches were connected to the skin of the forehead and back. The detailed circuitry connection is shown in Figure 2.

The AC voltage and current limiting output device are used to energize the rabbits in the AC group by the expert operator (Figure 2). The AC current was 2.1 mA. The AC voltage was 50~70 V with the frequency around 50 Hz. The trembling and contraction of the stimulated limb muscles can be seen during the process of treatment. The duration of AC treatment was 60~90 min (90 min on day 3, 60 min on day 7 and 14). Rabbits in the M group were placed in the same fixed frame as the AC group without treatment. We recorded no observable adverse effects—such as localized tissue burning, significant or painful muscle contractions, inflammation or skin damage at the electrode sites, or any signs of systemic stress (e.g., changes in feeding, drinking, or mobility behavior).

### 2.4. Bone Specimen Collection

Five to six rabbits were executed in the M and AC groups randomly at day 3, day 7, day 14, and day 28 after electrotherapy. Fractured radial bones were placed in 4% paraformaldehyde after washing with normal saline. The bones were decalcified with 20% EDTA for four weeks. Then, the samples were dehydrated with gradient ethanol and transparent xylene, and embedded in paraffin.

### 2.5. Histopathology and Immunohistochemistry

After fixation in 4% paraformaldehyde, joint tissues were decalcified with 10% EDTA solution (pH 7.2). After being embedded in paraffin, the tissues were sliced into 4 μm sections and stained with hematoxylin and eosin (HE) using standard procedures. Tissues were incubated overnight with an antibody against BMP-2 (1:100, Bioss, Beijing, China) and VEGF (1:200, Proteintech, Rosemont, IL, USA) at 4 °C to detect the contents of BMP-2 and VEGF in callus by immunohistochemistry. The staining intensity was measured by Image J software (1.53a).

### 2.6. X-Ray Evaluation

X-rays were performed to examine the bone fracture of the extracted radius on the day of execution. The fracture healing was scored by the Lane Sandhu X-ray scoring standard. The film results were scored by two imaging doctors with a double-blind method. The criteria of Lane Sandhu X-ray scoring included three aspects, including bone formation, bone connection, and bone shaping. The scoring criteria of bone formation, bone connection, and bone shaping referred to previous protocols [19].

### 2.7. Micro-CT Evaluation

Micro-CT (Skyscan1276 X-Ray Microtomograph, Bruker, Billerica, MA, USA) was used to evaluate the width and depth of bone fracture at day 3, day 7, and day 14. In addition, Micro-CT was also used to evaluate the actual structure of the bone cortex and bone trabecula so that two-dimensional and three-dimensional images could be obtained. The resolution of the image was 20 μM. NRecon (Version 1.7.4.6) software is used for three-dimensional image reconstruction. DataViewer, CTan, and CTvox software was used for three-dimensional analyses and mapping. The new bone formation in the fractured area was analyzed [20,21].

### 2.8. Cell Experiments

#### 2.8.1. Cell Culture and Electrical Stimulation

MC3T3-E1 (pre-osteoblastic cells) subclone 14 cells were acquired from Wuhan Pricella Biotechnology Co., Ltd. (Wuhan, China) and cultured in MEM-alpha medium supplemented with 10% FBS, with the medium being replaced every two days. For the electrical stimulation group, cells in 6-well plates were subjected to AC fields using parallel carbon rod electrodes immersed in the culture medium on opposite sides of each well. Using an IonOptix cell stimulation culture system, we applied a 50 Hz AC with a pulse width of 400 μs at a nominal electric field strength of 0.4 V/cm. The cells were examined daily under a microscope, and after 7 days of stimulation, the cells were collected for subsequent experiments.

#### 2.8.2. Alizarin Red S Staining

Alizarin Red S staining was performed by incubating the samples with 0.5% Alizarin Red S solution at room temperature for 20 min, followed by washing with deionized water to remove excess dye. The stained samples were then observed under 4× and 20× magnification, and quantitative analysis was conducted on the positive areas to calculate the percentage of the area stained.

#### 2.8.3. Alkaline Phosphatase (ALP) Activity Assay

The activity of alkaline phosphatase was assessed using an ALP activity assay kit (Beyotime, Shanghai, China) in accordance with the manufacturer’s instructions. For each sample, cell lysis was performed using lysis buffer. Samples were then allocated into a 96-well plate, categorized into blank controls, standards, and test samples, followed by the addition of a chromogenic substrate. The reaction mixture was incubated at 37 °C for 15 min before the addition of a stop solution to halt the reaction. Enzymatic activity was measured at a wavelength of 405 nm, and activity levels were compared with those of the control group.

### 2.9. RNA-Seq Analysis

Total RNA was extracted from cells or tissue samples, and Nanodrop2000 was used to detect the concentration and purity of the extracted RNA. Agarose gel electrophoresis was used to detect RNA integrity, and the RNA Integrity Number (RIN) value was determined with Agilent 2100. A single library construction requires total RNA ≥ 1 μg with the concentration ≥ 35 ng/μL, OD260/280 ≥ 1.8, and OD260/230 ≥ 1.0. The sequencing library of each RNA sample was prepared by using Ion total RNA-Seq Kit v2 (Life technologies, Carlsbad, CA, USA) according to the protocol provided by the manufacturer. The cDNA libraries were sequenced with an Illumina NovaSeq 6000 according to standard sequencing protocol.

### 2.10. Differential Genes Expression Analysis

Fast-QC software was used to assess the quality of the data, including the distribution of the base quality value, GC contents, the proportion of PCR duplication, and the frequencies of K-mer. Furthermore, we used Mapsplicing as our RNA-seq read mapping analysis tool to identify the exon-exon splicing immediately and accurately. Finally, we applied DEseq2 to filter the differentially expressed genes. After the statistical analysis, we screened the differentially expressed genes (DEGs) by the following criteria: fold change ≥ 2 or ≤0.5, *p*-value < 0.05.

### 2.11. Functional Enrichment Analysis

To investigate gene functions and uncover signaling pathways among the selected DEGs, GO analysis was applied to analyze the biological function of DEGs according to the GO database. Pathway analysis was used to identify the significant pathway of DEGs according to the KEGG database. A Fisher exact test and χ^2^ test were used to classify the GO terms. GO and pathway categories with *p*-value < 0.05 were selected for further analysis.

### 2.12. Multiplex Immunohistochemical Staining

According to the manufacturer’s instructions, multiple immunohistochemical experiments were performed using the TSAPLus Fluorescent Triple Staining Kit for tyramide signal amplification immunofluorescence (Servicebio, Wuhan, China, G1236), which employs Tyramide Signal Amplification (TSA) technology. In brief, 3 μm tissue sections from mouse ankle joints, fixed in formalin and embedded in paraffin, were deparaffinized with xylene and rehydrated through an ethanol gradient. Sections underwent microwave-induced antigen retrieval in tris-EDTA buffer (pH = 9.0) and were treated with 0.3% hydrogen peroxide in methanol to block endogenous peroxidase activity. The sections were washed in PBST and blocked with 5% goat serum in PBS for 10 min, followed by overnight incubation at 4 °C with primary antibodies. After additional washing in PBST, slides were developed using the horseradish peroxidase-labeled goat anti-rabbit secondary antibody, diluted in the signal amplification reagent provided by the kit. For multiple fluorescence staining, sections were processed starting from the antigen retrieval step to remove bound antibodies, and then incubated with another primary antibody until all antigens were stained. Finally, sections were counterstained with DAPI and mounted using a glycerol-gelatin mounting medium. Primary antibodies used included Bglap (GeneTex, San Antonio, TX, USA, GTX13418), Runx2 (bioss, bs-1134R), and Sp7 (bioss, bs-1110R).

### 2.13. Statistical Analysis

Data are presented as mean ± standard deviation (SD). The independent-sample *t* test and non-parametric test were used to analyze the Lane Sandhu X-ray scores, new bone volume fraction, and levels of BMP-2 and VEGF of the two groups. The gene sequencing data were analyzed on the Majorbio platform. *p*-values less than 0.05 were considered significant.

## 3. Results

### 3.1. X-Ray Scanning of Bone Fracture

**Figure 3:**
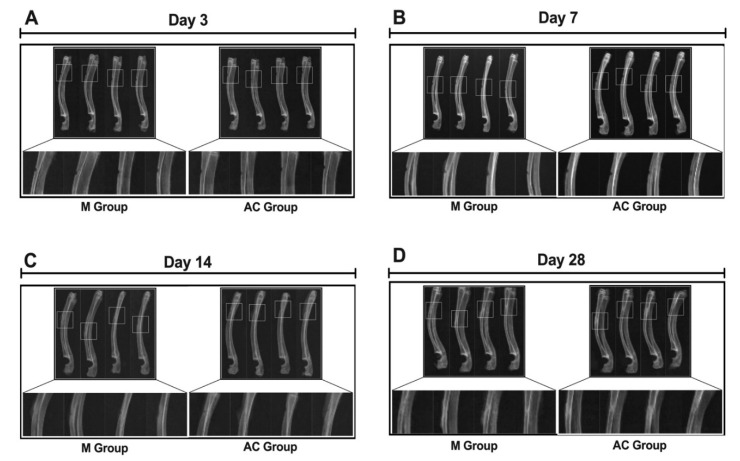
X-ray scanning of bone defect. (**A**) X-ray scanning image of bone defect (day 3); (**B**) X-ray scanning image of bone defect (day 7); (**C**) X-ray scanning image of bone defect (day 14); (**D**) X-ray scanning image of bone defect (day 28).

X-ray scanning was used to evaluate bone formation, bone shaping, and bone connection in the fractured bone. In bone formation and shaping, a significant difference between the alternating current group (AC group) and model group (M group) (shown in Figure 3 and Table 1) was observed. In bone formation, the score of the AC group (3.40 ± 0.55) was significantly higher than that of the M group (2.70 ± 0.27) on day 14 after treatment (*p* < 0.05). In bone shaping, the score of the AC group (3.00 ± 1.00) was also significantly higher than that of the M group (1.60 ± 0.89) on day 14 after treatment (*p* < 0.05). The results of X-ray evaluation indicated that AC stimulation was demonstrated to have a beneficial effect on bone formation and shaping of a fractured bone.

### 3.2. Micro-CT Scanning of Bone Fracture

**Figure 4:**
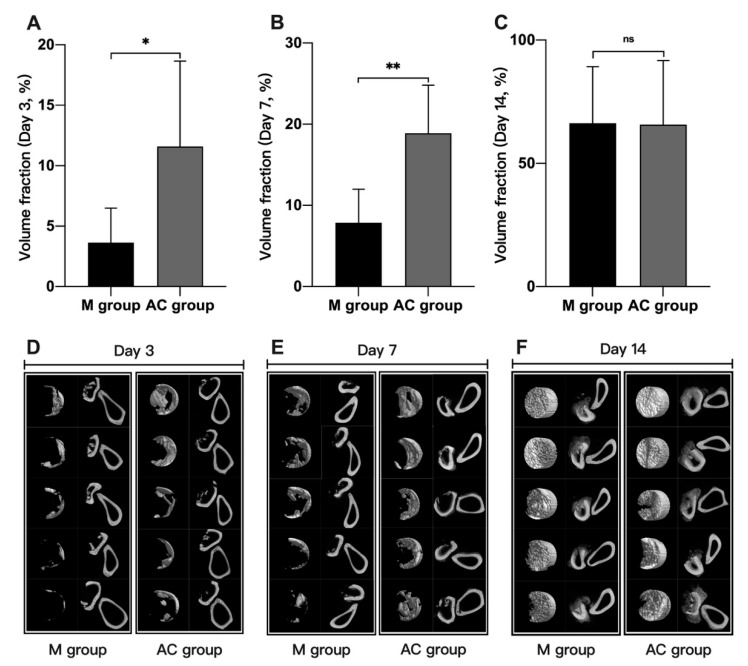
Micro-CT scanning results of new bone in defect (n = 5~6). (**A**) Comparison of new bone volume fraction in M and AC group at day 3 after treatment; (**B**) Comparison of new bone volume fraction in M and AC group at day 7 after treatment; (**C**) Comparison of new bone volume fraction in M and AC group at day 14 after treatment; (**D**) Micro-CT scanning image of new bone in defect (day 3); (**E**) Micro-CT scanning image of new bone in defect (day 7); (**F**) Micro-CT scanning image of new bone in defect (day 14); M = model group, AC = alternating current. ns: no significance, * *p* < 0.05, ** *p* < 0.01. Left line: new bone in bone defect. Right line: transverse scanning images of bone defect.

Micro-CT was used to measure the accurate fraction volume of new bone in a fractured bone. On day 3, the fraction volume of the AC group (11.58% ± 7.07%) was higher than that of the M group (3.63% ± 2.87) (Figure 4). On day 7, the fraction volume of AC group (18.90% ± 5.92%) was higher than that of M group (7.87% ± 4.13%). The results of Micro-CT showed that AC stimulation could promote the formation of new bone in the early stage of fractured bone healing.

### 3.3. HE Staining of Fractured Bone Healing

**Figure 5:**
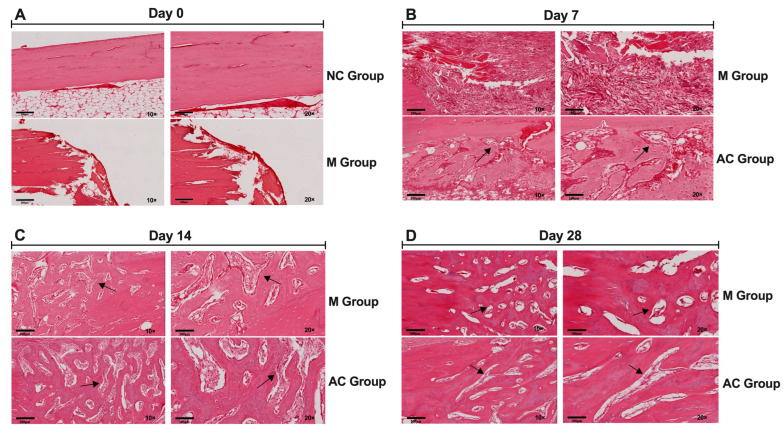
Morphological observation of bone defect by HE staining. (**A**) HE staining of bone defect on day 0 in NC and M group. (**B**) HE staining of bone defect on day 7 after treatment in M and AC group. (**C**) HE staining of bone defect on day 14 after treatment in M and AC group. (**D**) HE staining of bone defect on day 28 after treatment in M and AC group. On day 7, a small amount of trabecular bones (black arrow) were observed in AC group, while rare trabecular bone was observed in M group. On day 14, a large number of mature trabecular bones (black arrow) were observed in AC group, while less trabecular bone (black arrow) was observed in M group. On day 28, there were no differences in morphology between M and AC group. NC = normal group, M = model group, AC = alternating current.

HE staining was used to evaluate morphological changes at day 7, 14, and 28 after AC treatment. As shown in Figure 5, at day 7 after AC treatment, the AC group has a small amount of trabecular bone formation around the bone fracture. The M group had less trabecular bone formation at day 7. Some fibrous connective tissues were observed in the M group. On day 14, a large number of mature trabecular bone formations were found around the fractured site in the AC group. The trabecular bones were numerous and arranged neatly in the AC group. The M group had less trabecular bone formation at day 14. Trabecular bone formation was observed around the bone-fractured site in the M groups. The arrangement of trabecular bones was sparse and less than in the AC group. Thus, the fractured bone healing process was significantly promoted in the AC group. On day 28, the matured trabecular bone and plate-like bone formation were filled in the M and AC groups. There were no differences in morphology in the two groups.

### 3.4. Immunohistochemistry of BMP-2 and VEGF in Bone Fractures

**Figure 6:**
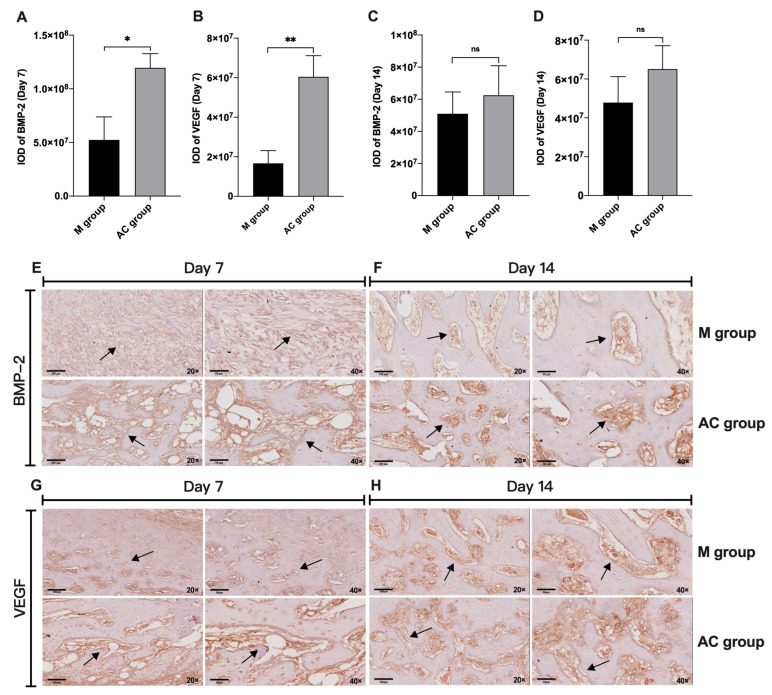
Expression of BMP-2 and VEGF in bone defect. (**A**) Histogram of BMP-2 expression with M and AC groups on day 7 after treatment. (**B**) Histogram of VEGF expression with M and AC groups on day 7 after treatment. (**C**) Histogram of BMP-2 expression with M and AC groups on day 14 after treatment. (**D**) Histogram of VEGF expression with M and AC groups on day 14 after treatment. (**E**,**F**) Immunohistochemical staining of BMP-2 in bone defect of M and AC group. (**G**,**H**) Immunohistochemical staining of VEGF in bone defect of M and AC group. ns: no significance, * *p* < 0.05, ** *p* < 0.01. Black arrows: positive staining area.

The expression of BMP-2 and VEGF in fractured bone on day 7 and 14 after treatment were evaluated, as shown in Figure 6, since the treated effects showed significant difference in these two treatment times. For BMP-2, the AC group was higher than the M group on day 7 after AC treatment, but there was no difference in BMP-2 between the two groups on day 14 after AC treatment. For VEGF, the AC group was higher than the M group on day 7 after treatment, but there was no difference in VEGF between the two groups on day 14 after AC treatment.

### 3.5. In Vitro Experiments of AC on Osteogenic Differentiation

**Figure 7:**
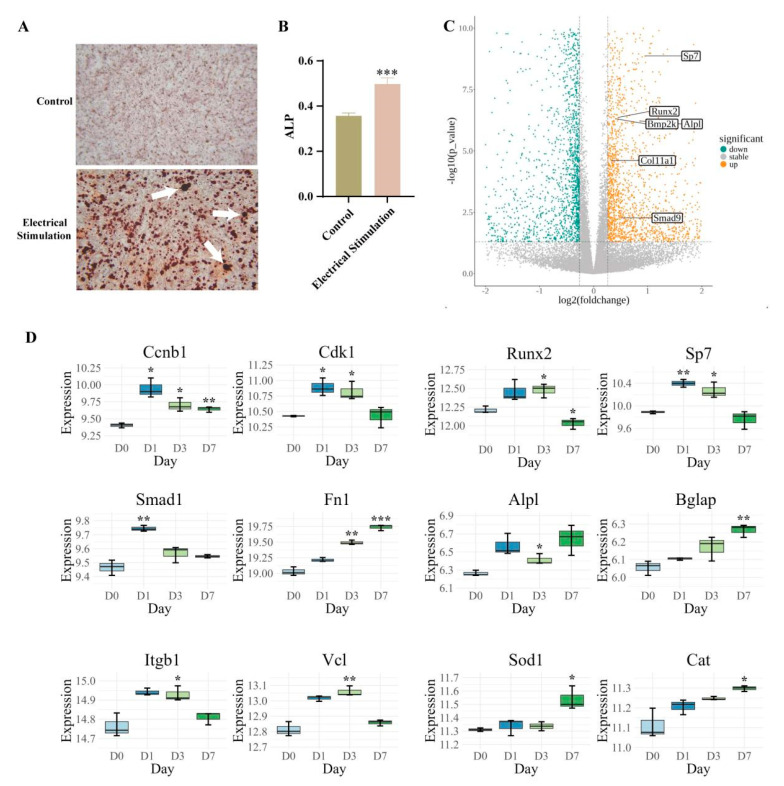
Effects of electrical stimulation on osteogenic differentiation. (**A**) Alizarin Red S staining demonstrating calcium deposits at 4× magnification as indicators of osteogenic differentiation in control and electrical stimulation groups. Arrow indicated calcified nodules. (**B**) Quantitative analysis of alkaline phosphatase (ALP) activity, showing a significant increase in the electrical stimulation group in comparison to the control group. (**C**) Volcano plot illustrating a broad spectrum of gene expression alterations, with significant upregulation noted in genes pertinent to osteogenic differentiation. (**D**) Box plots representing the expression variations of genes at different time points (day 0, day 1, day 3, and day 7). Statistical significance for gene expression is indicated as follows: * *p* < 0.05, ** *p* < 0.01, *** *p* < 0.001. All statistical differences are compared to the day 0 group.

In order to reveal the molecular mechanism, pre-osteoblastic cells were subjected to electrical stimulation under the same conditions (electric field strength of 0.4 V/cm, frequency of 50 Hz, and pulse width of 400 µs AC) as the AC treatment to observe if the osteogenic differentiation could happen. With this study, we found that AC could indeed stimulate osteogenic differentiation of pre-osteoblastic cells (MC3T3-E1 subclone 14 cells), as indicated by Alizarin Red S staining in the electrical stimulation group compared to controls, with distinct calcified nodules visible at 4× magnification (Figure 7A). Quantitative analysis further supported these findings, showing a significant increase in alkaline phosphatase (ALP) activity in the electrical stimulation group (Figure 7B). Subsequently, samples were collected for RNA sequencing at various time points, day 0, day 1, day 3, and day 7, following the application of electrical stimulation to the cells. Gene expression analysis via a volcano plot revealed a broad spectrum of gene changes, particularly noting significant upregulation of genes critical to osteogenic differentiation in the electrically stimulated samples (Figure 7C). Temporal expression patterns of proliferation-related genes such as Ccnb1 and Cdk1 were monitored at multiple time points (day 0, day 1, day 3, and day 7), with box plots demonstrating marked expression variations under electrical stimulation (Figure 7D). The expression of key osteogenic initiation factors (Runx2, Sp7, and Smad1) and extracellular matrix-related genes (Fn1) was analyzed at the same time points, showing trends that promote osteogenic differentiation and enhance bone matrix synthesis and cell–matrix interactions, respectively. Mineralization genes (Alpl and Bglap) were also differentially expressed, marking the transition into the mineralization phase of osteogenic differentiation. Furthermore, the expression profiles of cell adhesion and migration-related genes, such as Itgb1 and Vcl, were examined. Box plots highlighted their enhanced expression at different time points (day 0, day 1, day 3, and day 7) in the electrically stimulated group, illustrating their crucial roles in promoting cell adhesion and positioning during osteogenic differentiation. Additionally, the gene expression of antioxidants and stress response proteins, including Sod1 and Cat, was investigated. Their upregulation under electrical stimulation, as shown in the box plots, indicates an active cellular defense mechanism against oxidative stress, contributing to the overall resilience and viability of osteogenic cells. These findings collectively underscore the multifaceted impact of electrical stimulation on enhancing osteogenic differentiation and bone tissue formation.

**Figure 8:**
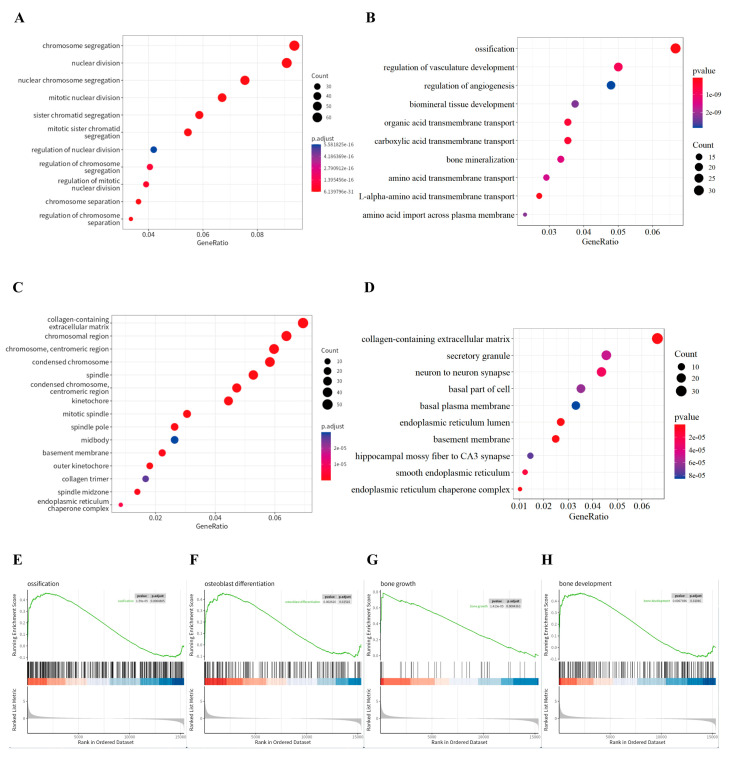
The impact of electrical stimulation on cellular pathways related to osteogenic differentiation. (**A**) GO (Gene Ontology) Biological Process (BP) pathway enrichment analysis comparing differentially expressed genes from day 3 to day 0. (**B**) GO Biological Process (BP) pathway enrichment analysis comparing differentially expressed genes from day 7 to day 0. (**C**) GO Cellular Component (CC) pathway enrichment analysis comparing differentially expressed genes from day 3 to day 0. (**D**) GO Cellular Component (CC) pathway enrichment analysis comparing differentially expressed genes from day 7 to day 0. Gene set enrichment analyses (**E**) for ossification, (**F**) osteoblast differentiation, (**G**) bone growth, and (**H**) bone development emphasize the biological processes and pathways enhanced by electrical stimulation, demonstrating enriched gene sets correlated to bone tissue growth and development.

Gene Ontology (GO) enrichment analysis was conducted on differentially expressed genes by comparing the results to day 0. The analysis of Biological Process (BP) pathways revealed a significant upregulation of genes associated with cell proliferation-related pathways, such as chromosome separation on day 3 (Figure 8A), and ossification pathways on day 7, illustrating the progression of osteogenic differentiation (Figure 8B). Further analysis of the Cellular Component (CC) pathways showed an upregulation of extracellular matrix-related pathways on both day 3 and day 7 (Figure 8C,D). This indicates that electrical stimulation enhances the synthesis of bone matrix and strengthens cell–matrix interactions, which are crucial for osteogenic differentiation. These findings highlight the critical pathways influenced by electrical stimulation at the early and late stages of cellular response, elucidating the dynamics of osteogenic differentiation under the electrical stimulated conditions. Gene set enrichment analyses further highlighted significant enhancements in biological processes and pathways related to ossification, osteoblast differentiation, bone growth, and bone development, with these processes being notably enriched in the electrically stimulated group (Figure 8E–H). Overall, the results demonstrate that electrical stimulation robustly promotes osteogenic differentiation by enhancing gene expression related to cell proliferation, matrix formation, and mineralization.

**Figure 9:**
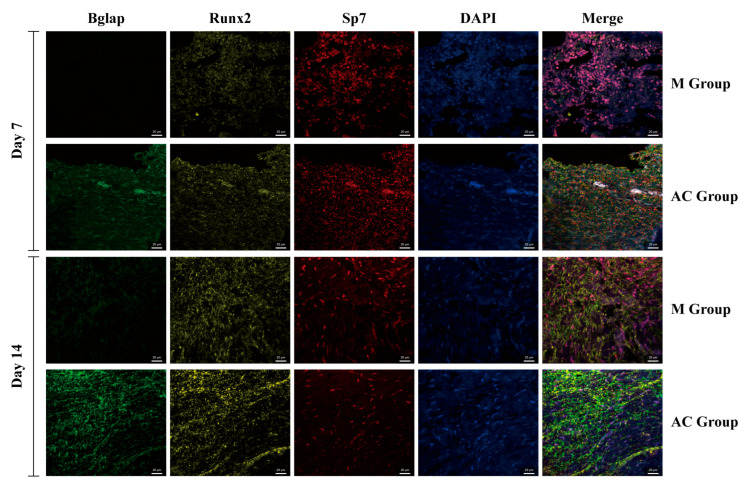
Multiple immunohistochemical staining of rabbit radial bone tissue sections.

Following multiple immunohistochemical staining of rabbit radial bone tissue sections, we observed significant increases in the expression of Bglap, Runx2, and Sp7 at the fracture sites after AC intervention, consistent with RNA-seq results (Figure 9). Notably, no elevation in Bglap expression was observed in the model group on either day 7 or day 14, indicating the absence of bone mineralization within 14 days in this group. In contrast, a significant increase in Bglap expression was detected on day 14 in the AC group, suggesting that AC intervention could potentially accelerate the process of bone mineralization. Additionally, expressions of Runx2 and Sp7 were also observed to increase. It is worth noting that the expression of Sp7 in the AC group decreased from day 7 to day 14, which may be due to Sp7 being an early marker of osteogenic differentiation, while Runx2 maintained a consistently high expression.

## 4. Discussion

In this study, we found that alternating current could significantly promote fractured bone healing, especially in the early stage. Alternating current could stimulate bone healing via bone formation and shaping, which were molecularly enhanced by the changes in gene expression after AC treatment.

Traumatic fractures lead to a substantial burden on healthcare systems worldwide. The emergency treatment of accelerating fracture healing has always been an important issue in orthopedics research [22,23]. In 1812, Harshone first used electric shocks to treat bone nonunion, which proved the effect of electrotherapy. In 1957, Fukada reported the phenomenon of the piezoelectric effect observed in the femur of man and ox [24]. They provided an electrophysiological explanation for electrotherapy to promote fracture healing. Electrical stimulation has gradually become a technique to promote fracture healing [25]. Nowadays, the existing forms of electrotherapy for skin wound healing contain direct current and unidirectional pulse current, constant direct current, pulsed direct current, pulsed electromagnetic field, and alternating current [26]. The parameters of electrical stimulation for fractured bone healing have always been the key point of electrical stimulation therapy [27].

The present study provided the first-ever evidence for the accelerated healing effect of alternating current on fractured bones, as demonstrated in a rabbit radial bone fracture model. From our observation, alternating current stimulation was an effective therapy for promoting fractured bone healing, especially in the early stage of fractured bone healing, suggesting the significant importance of alternating current for quickly healing fractured bones. For electrotherapies by alternating current stimulation, many attempts have been made for psychiatric and neurological disorders [28,29,30]. The waveforms, current sizes, and frequencies could cause different effects that promote fracture healing [31]. Among non-invasive approaches, extracorporeal shockwave therapy (ESWT) has demonstrated considerable clinical success, utilizing high-energy acoustic waves to stimulate osteogenesis. However, ESWT application can be associated with patient discomfort and requires specialized equipment [32]. Among electrical stimulation modalities, direct current (DC) has shown efficacy in promoting osteogenesis [33,34,35,36] but suffers from inherent limitations including electrode corrosion, tissue damage, and the creation of unfavorable pH microenvironments due to charge accumulation [37]. Capacitive coupling (CC) and pulsed electromagnetic field (PEMF) therapies circumvent some electrode-related issues but face challenges with inconsistent field distribution in complex bone geometries and limited penetration depth [38,39,40]. In contrast, AC stimulation offers a fundamentally different approach that addresses these limitations. The periodic reversal of current direction in AC prevents net charge accumulation, thereby eliminating the risks of electrode corrosion and tissue damage [41,42]. This feature enables safer and more sustained application. Compared to CC and PEMF, AC provides more uniform and controllable electric field distribution within bone tissue, ensuring consistent stimulation across the fracture site. This systematic comparison underscores the distinct advantages and innovation of our AC stimulation paradigm.

The therapeutic applications of electrical stimulation extend beyond local tissue regeneration to include the management of chronic pain through spinal cord mechanisms and the potentiation of systemic immune responses [43,44,45,46]. The cell experiments in this study demonstrated that AC could stimulate osteogenic differentiation of pre-osteoblastic cells, as indicated by the enhanced expression of related genes, explaining the mechanism of fast healing of fractured bone at the molecular level. Ccnb1 and Cdk1 were related with the fidelity of mitosis to stimulate the cell division cycle [47]. The Ccnb1/Cdk1 complex enhanced overall mitochondrial respiration during the G2/M transition, serving as a crucial coordinator of mitochondrial bioenergetics to facilitate the progression of G2/M cell division. Our study found Ccnb1 and Cdk1 were higher on day 1 and day 3 compared with day 0, indicating that AC could stimulate the cell division cycle in the early stage of fractured bone healing. Runx2 and Sp7 were involved in the development and maintenance of bone and cartilage related with osteoblast differentiation [48,49]. Smad1 mediated the signals of the bone morphogenetic proteins (BMPs), which were involved in osteogenesis [50]. Fn1 was an extracellular matrix protein that was upregulated during wound healing [51]. In our study, the changes in osteogenic initiation factors (Runx2, Sp7, and Smad1) and extracellular matrix-related genes (Fn1) indicated that AC could promote osteogenic differentiation and enhance bone matrix formation. Alpl encoded alkaline phosphatase, which plays a role in bone mineralization [52]. Bglap could encode a highly abundant bone protein secreted by osteoblasts to regulate bone remodeling [53]. The increase in Alpl and Bglap after AC treatment showed that mineralization was accelerated by AC treatment. Itgb1 forms integrin complexes that function as collagen receptors involved in cell adhesion [54]. Vcl was a cytoskeletal protein associated with cell–cell and cell–matrix junctions [55]. The changes in Itgb1 and Vcl observed in our study showed that AC could mediate cell adhesion and migration during fractured bone healing. Sod1 catalyzed the disproportionation of superoxide to hydrogen peroxide and dioxygen [56]. Cat was a key antioxidant enzyme to mitigate the toxic effects of hydrogen peroxide [57]. The increase in Sod1 and Cat after AC treatment indicated that AC can boost the function against oxidative stress to the viability of osteogenic cells. The GO and KEGG analysis of molecular pathways also indicated that AC could induce osteogenic differentiation, synthesis of bone matrix, cell–matrix interactions, bone growth, and bone development.

The present study clearly indicated the important clinical significance of AC treatment in the rehabilitation of bone fractures. Bone fractures are one of the most common traumatic injuries to humans [58]. The long period of bone fracture rehabilitation can impact the quality of life of patients, leading to disability and depression. Spending a long time bedridden or experiencing joint fixation can also lead to severe cardiovascular events and pulmonary embolism [59]. In addition, the direct costs for individuals and society correlated with bone hospitalization and rehabilitation are enormous [60,61,62]. By accelerating the recovery of bone fractures, AC could improve the quality of life in bone fracture patients. Furthermore, the direct costs for individuals and society caused by bone fractures could be reduced by AC treatment. The optimization of AC parameters including current, voltage, and frequency should be explored to benefit bone fracture patients in the future, when the AC device can also be developed for clinical application.

## 5. Summary and Outlook

This study demonstrates that alternating current (AC) stimulation significantly promotes bone fracture healing through multi-level experimental validation. Using integrated in vivo and in vitro models, we found that AC treatment enhances early-stage fracture repair by promoting osteogenic differentiation, accelerating bone matrix formation, and upregulating key osteogenic factors including BMP-2 and VEGF. Radiographic, histological, and transcriptomic analyses consistently confirmed AC’s ability to modulate molecular pathways critical to bone regeneration. These findings establish AC stimulation as a promising non-invasive therapeutic strategy for fracture management, providing new insights into bioelectrical approaches for bone repair.

While this study provides valuable insights into AC stimulation for bone fracture healing, several limitations should be addressed in future research. Parameter optimization represents a crucial next step to maximize therapeutic efficacy while ensuring patient comfort and minimizing adverse sensations. Subsequent studies should also incorporate more comprehensive evaluation methods, including advanced imaging techniques and functional assessments, to provide deeper insights into bone formation and healing effects. Additionally, establishing clear safety thresholds through the systematic monitoring of tissue responses will be essential for clinical implementation. Through continued investigation along these strategic directions, AC stimulation holds strong promise to evolve into a safe and effective therapeutic approach that can significantly improve fracture recovery and enhance patient quality of life.

## Figures and Tables

**Table 1:** Lane Sandhu X-ray score of fracture healing (x ± s) (n = 5~6).

Group	Bone Formation				Bone Connection				Bone Shaping			
	Day 3	Day 7	Day 14	Day 28	Day 3	Day 7	Day 14	Day 28	Day 3	Day 7	Day 14	Day 28
M	1.75 ± 0.42	1.40 ± 0.42	2.70 ± 0.27	4.00 ± 0	1.50 ± 0.84	1.00 ± 0	2.40 ± 0.89	4.00 ± 0	1.17 ± 0.98	0.40 ± 0.55	1.60 ± 0.89	4.00 ± 0
AC	1.92 ± 0.49	1.90 ± 0.82	3.40 ± 0.55 *	4.00 ± 0	2.33 ± 0.52	0.40 ± 0.89	2.80 ± 0.84	4.00 ± 0	1.33 ± 0.82	0.40 ± 0.89	3.00 ± 1.00 *	4.00 ± 0

Note: * compared with the M group, *p* < 0.05.

## Data Availability

The data that support the findings of this study are available from the corresponding author upon reasonable request.
